# Supplementary material for: Self-powered H2 production with bifunctional hydrazine as sole consumable
Source: Nat Commun. 2018 Oct 19;9:4365. doi: 10.1038/s41467-018-06815-9 (PMC6195518; doi:10.1038/s41467-018-06815-9)
Supplement: Supplementary file 1 — Supplementary Information [file 41467_2018_6815_MOESM1_ESM.pdf]

Supplementary Information

Self-powered H_2 production with bifunctional hydrazine as sole consumable

By Liu et al.

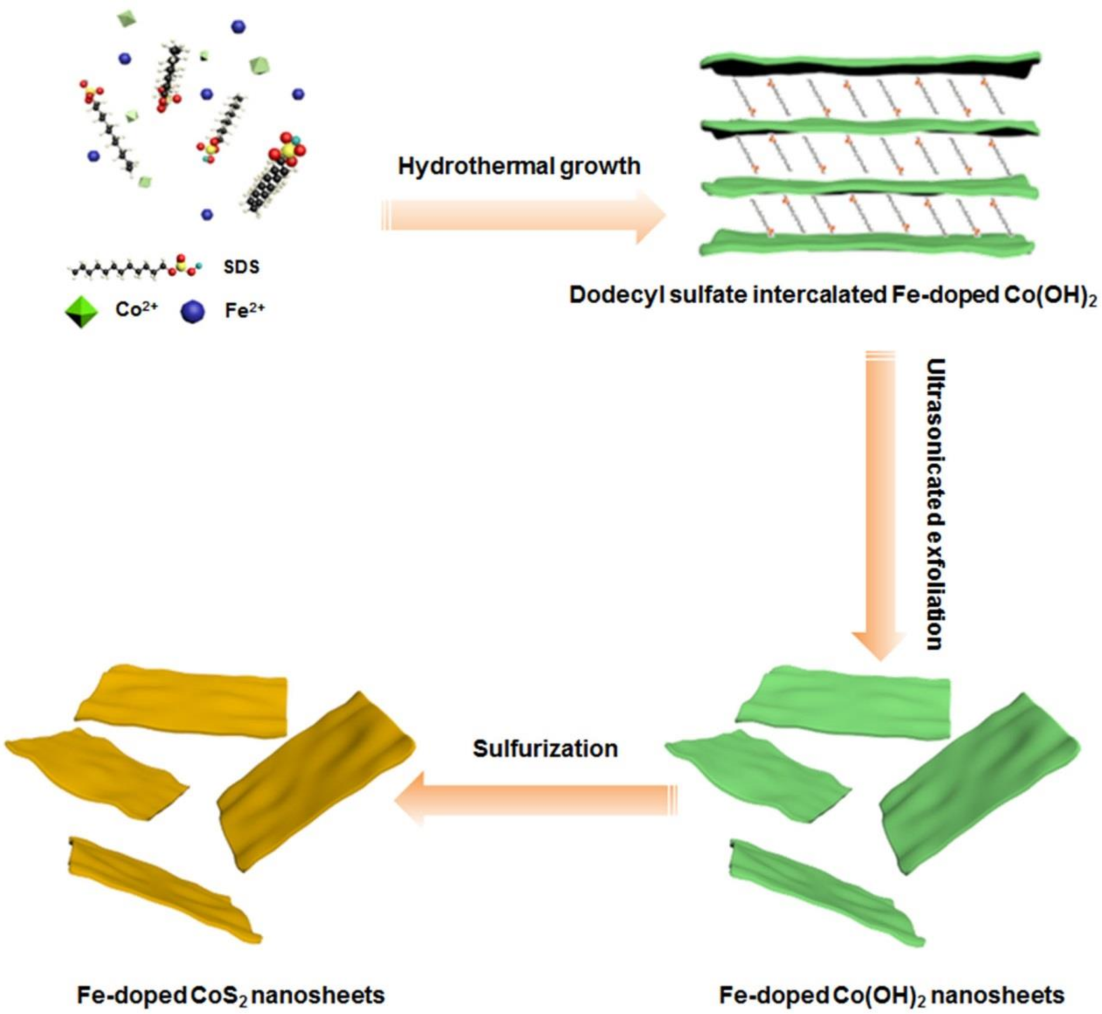

Supplementary Figure 1 | Schematic illustration of the preparation procedure of the Fe-doped CoS_2 nanosheets. SDS is sodium dodecyl sulfate. When no Fe^{2+} ions were introduced, pure $Co(OH)_2$ and then pure CoS_2 nanosheets were obtained.

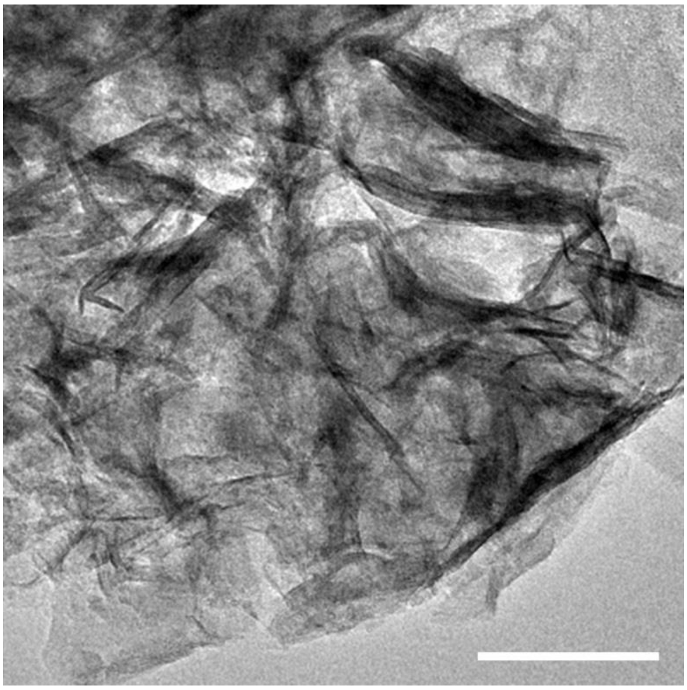

Supplementary Figure 2 | TEM image of the Fe-doped $Co(OH)_2$ nanosheets. These Fe-doped $Co(OH)_2$ nanosheets are those mentioned in Supplementary Fig. 1. Scale bar, 50 nm.

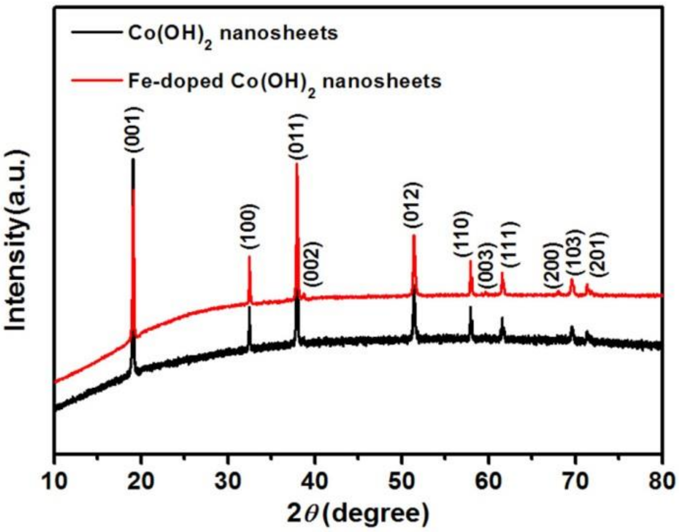

Supplementary Figure 3 | XRD patterns of the Fe-doped and the pure $Co(OH)_2$ nanosheet precursors. Both of the two patterns can be unambiguously indexed to the standard $Co(OH)_2$, according to No. 74-1057 Powder Diffraction File (PDF). This indicates that the Fe content is low enough not to disturb the $Co(OH)_2$ crystal lattice of the Fe-doped $Co(OH)_2$.

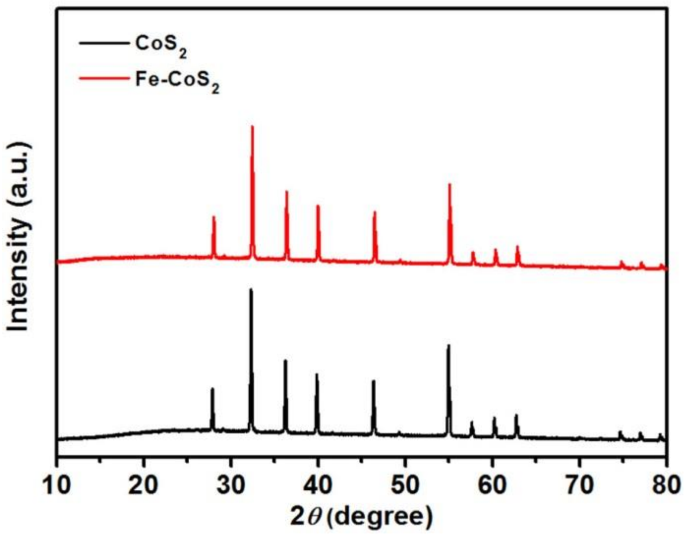

Supplementary Figure 4 | XRD patterns of the pure CoS_2 nanosheets and the Fe-CoS_2 ones, which are identical. Note that the red pattern here is the same one as the black one in Fig. 1a.

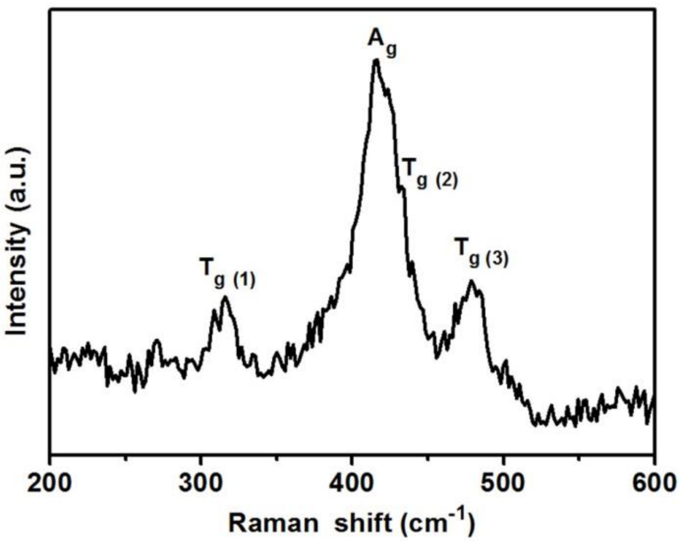

Supplementary Figure 5 | Raman spectrum of the Fe-CoS_2 nanosheets. The Raman bands at 314, 415, 435 and 478 cm^{-1} can be assigned to the $T_{g(1)}$, A_g, $T_{g(2)}$ and $T_{g(3)}$ vibrational modes of CoS_2 (ref. 1).

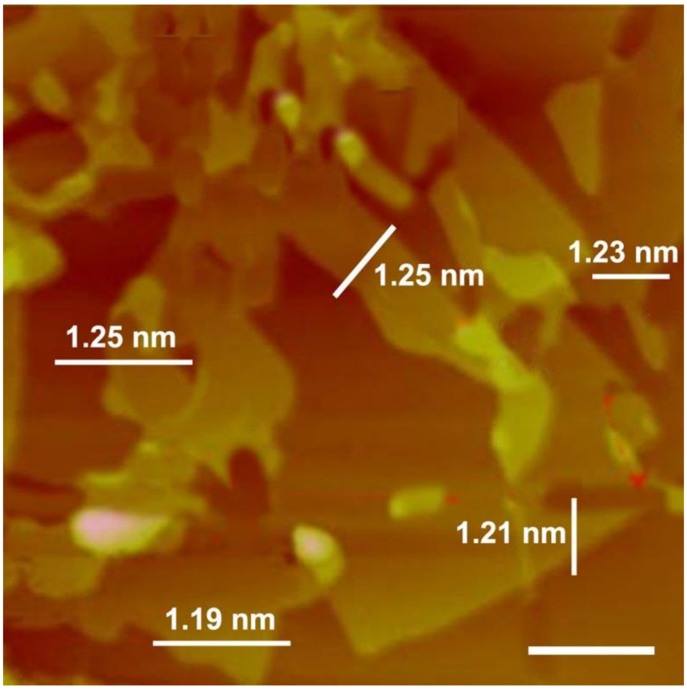

Supplementary Figure 6 | Additional AFM image of the Fe-CoS_2 nanosheets. The thickness values here, along with the four in Fig. 1c, d, give the average thickness and standard deviation to be 1.22 and 0.03 nm, respectively. Scale bar, 50 nm.

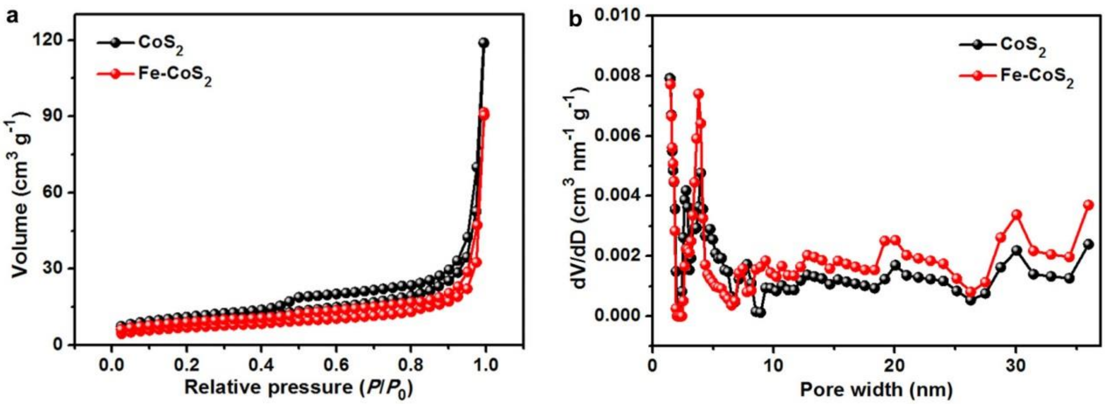

Supplementary Figure 7 | N_2-sorption isotherms and pore size distribution curves of the CoS_2 nanosheets and the Fe-CoS_2 ones. a N_2-sorption isotherms. **b** Pore size distribution curves. P is the pressure of N_2 in the sample cell of the analyzer instrument (see more details in Methods) when equilibrium is achieved, and P_0 is the saturation pressure of N_2 at the analysis temperature. The isotherms give the specific surface area values of CoS_2 and Fe-CoS_2 to be 25.3 and 27.2 $m^2\ g^{-1}$, respectively. The two values are close to each other. The pore size distribution curves indicate the presence of micropores (<2 nm) and mesopores (2~50 nm) in both of CoS_2 and Fe-CoS_2. These findings confirm that the Fe doping did not change the morphology and structure of the nanosheets, which is consistent with the XRD (Fig. 1a and Supplementary Fig. 4), Raman (Supplementary Fig. 5), and HRTEM (Fig. 1f) results.

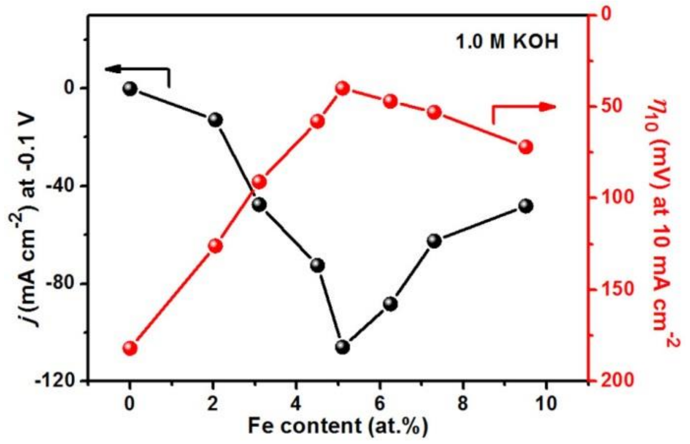

Supplementary Figure 8 | Dependences of the HER electrocatalytic current density (*j*) and overpotential (*η*) of the Fe-CoS$_2$ catalyst on the atomic percentage of Fe. The *j* and *η* values were measured in 1.0 M KOH at the working potential of – 0.1 V and the current density of 10 mA cm^{-2}, respectively. η_{10} denotes *η* measured at the current density of 10 mA cm^{-2}. Larger *j* and smaller *η* values indicate higher HER activities.

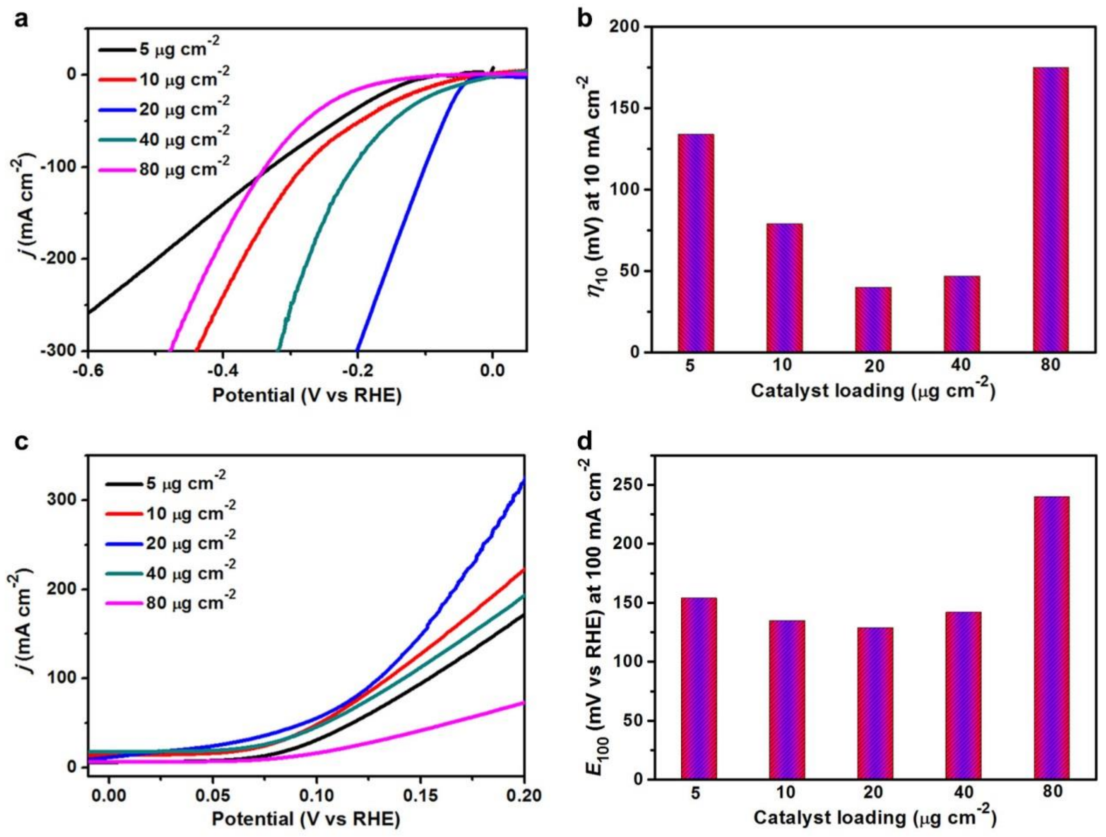

Supplementary Figure 9 | HER and HzOR performances of the Fe-CoS$_2$ nanosheets at different loadings. a, **b** HER polarization curves at different loadings (**a**) and the corresponding required η values for 10 mA cm^{-2} (**b**) , in which η for 10 mA cm^{-2} is denoted as η_{10}. **c**, **d** have the same meanings as **a**, **b** but for HzOR. E_{100} has the same meaning as that in the main text, which means the potential for 100 mA cm^{-2}. Note that the blue curves in Fig. 2a, d are redrawn here as the blue ones in **a** and **c**, respectively. The measurement conditions of **a** and **c** are the same as those of Fig. 2a, d, respectively. The results here show that when the loading increased, the η_{10} or E_{100} value initially decreased, reached the lowest point at 20 µg cm^{-2} and then increased. These changes indicate that the HER and the HzOR performances of Fe-CoS$_2$ were the highest at 20 µg cm^{-2}. Thus, we used this loading for the HER and the HzOR experiments in the main text. In addition, the increase of η_{10} or E_{100} after 20 µg cm^{-2} was possibly due to the slow electron-transfer kinetics caused by the over-loading of catalysts[2].

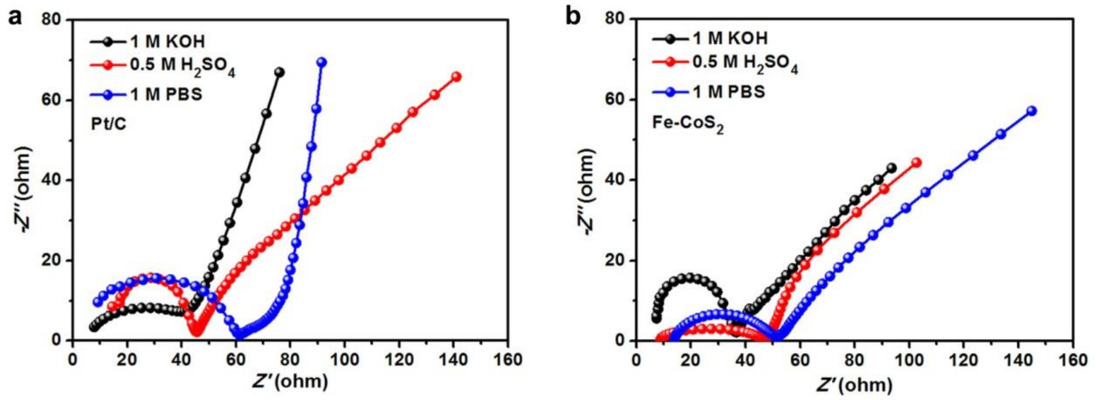

Supplementary Figure 10 | EIS plots at the applied potential of – 0.25 V. a EIS plots of 20 wt.% Pt/C. **b** EIS plots of the Fe-CoS_2 nanosheets. The EIS spectra give the charge transfer resistance (R_{ct}) values of Fe-CoS_2 to be 24.2 Ω (1.0 M KOH), 35.5 Ω (1.0 M PBS) and 39.2 Ω (0.5 M H_2SO_4) and those of Pt/C to be 47.3 Ω (1.0 M KOH), 56.4 Ω (1.0 M PBS) and 25.8 Ω (0.5 M H_2SO_4).

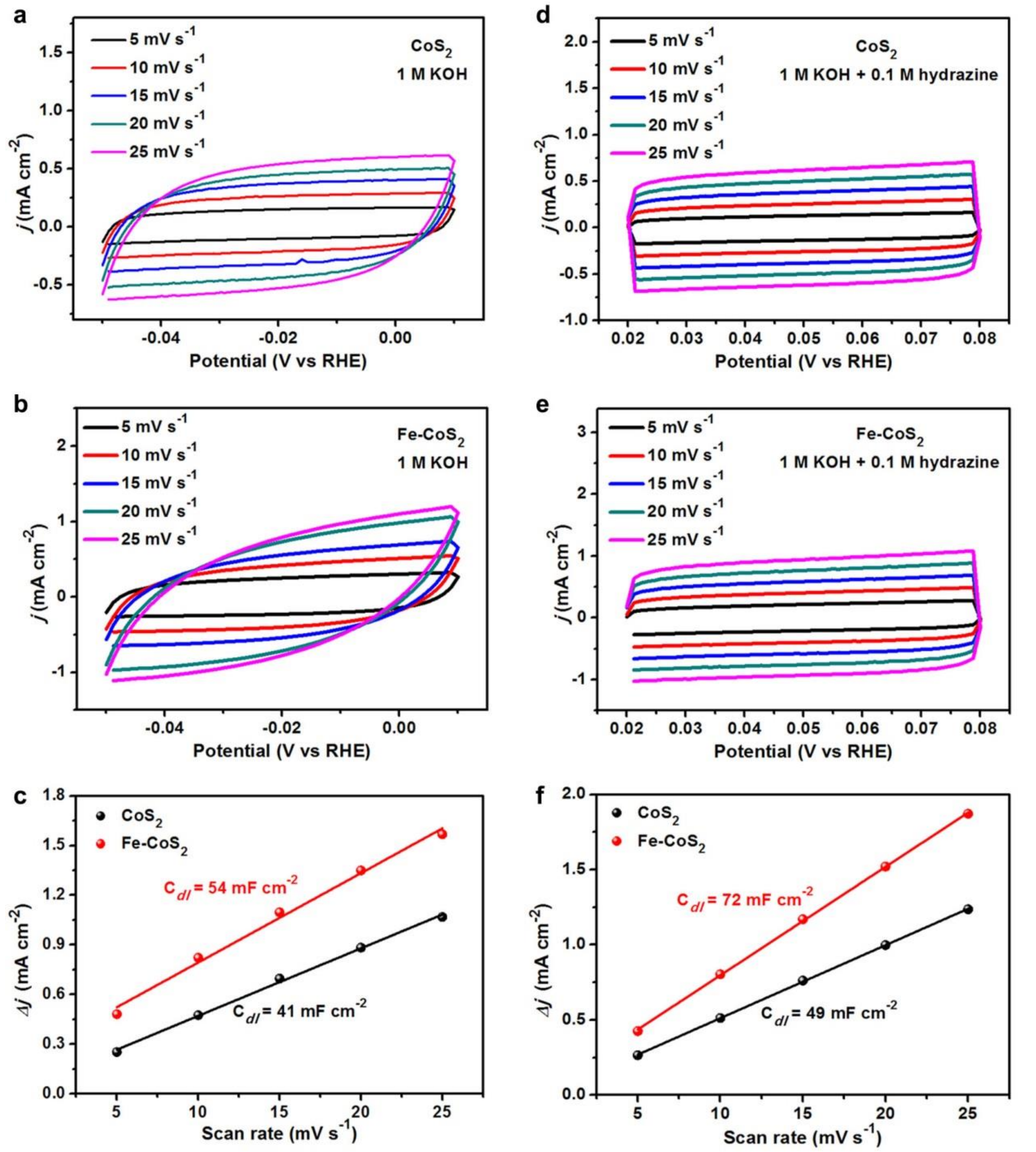

Supplementary Figure 11 | C_{dl} measurements corresponding to the HER and the HzOR experiments. a, **b** Cyclic voltammogram (CV) curves at the scan rates of 5, 10, 15, 20 and 25 mV s^{-1} in the potential range without Faradaic processes for measuring C_{dl} of the CoS_2 nanosheets and the Fe-CoS_2 ones. The other measurement conditions are the same as those of the HER measurements in Fig. 2a. **c** Capacitive current (*Δj*) at – 0.02 V against the scan rate, in which the labeled C_{dl} values were

obtained by the linear fittings on the plots. The Δj values are from the curves in **a**, **b**. **d**–**f** have the same meanings as **a**–**c** but are corresponding to the HzOR measurements in Fig. 2d. The working potential corresponding to the Δj values in (f) is 0.05 V. By the C_{dl} values, the ECSA values of CoS_2 and Fe-CoS_2 were derived[3–5] to be 683 and 900 cm^2, respectively, for the HER, and 817 and 1,200 cm^2, respectively, for the HzOR. The two pairs of ECSA values indicate the presence of more active surface areas on Fe-CoS_2 than on CoS_2, in a good agreement with the better HER and HzOR activities of Fe-CoS_2 than CoS_2 (Fig. 2a, d).

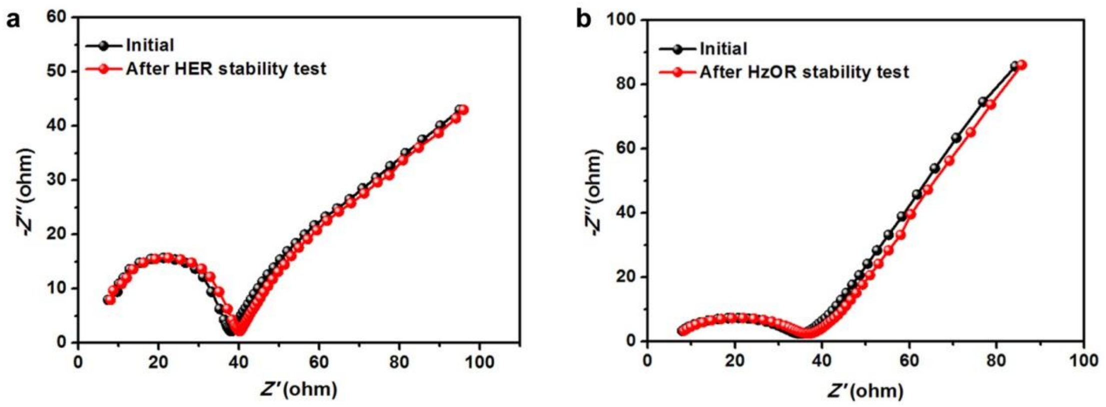

Supplementary Figure 12 | EIS plots of the Fe-CoS_2 nanosheets before and after the chronoamperometric measurements (namely the HER- and HzOR-stability experiments in Fig. 2c, f) at an open-circuit potential. a EIS plots before and after the HER chronoamperometric measurement. **b** EIS plots before and after the HzOR chronoamperometric measurement. The two spectra before and after the HER chronoamperometric stability test are almost identical to each other. So are the two before and after the HzOR chronoamperometric stability test. These results indicate that Fe-CoS_2 has stable electrocatalytic kinetics through the stability tests.

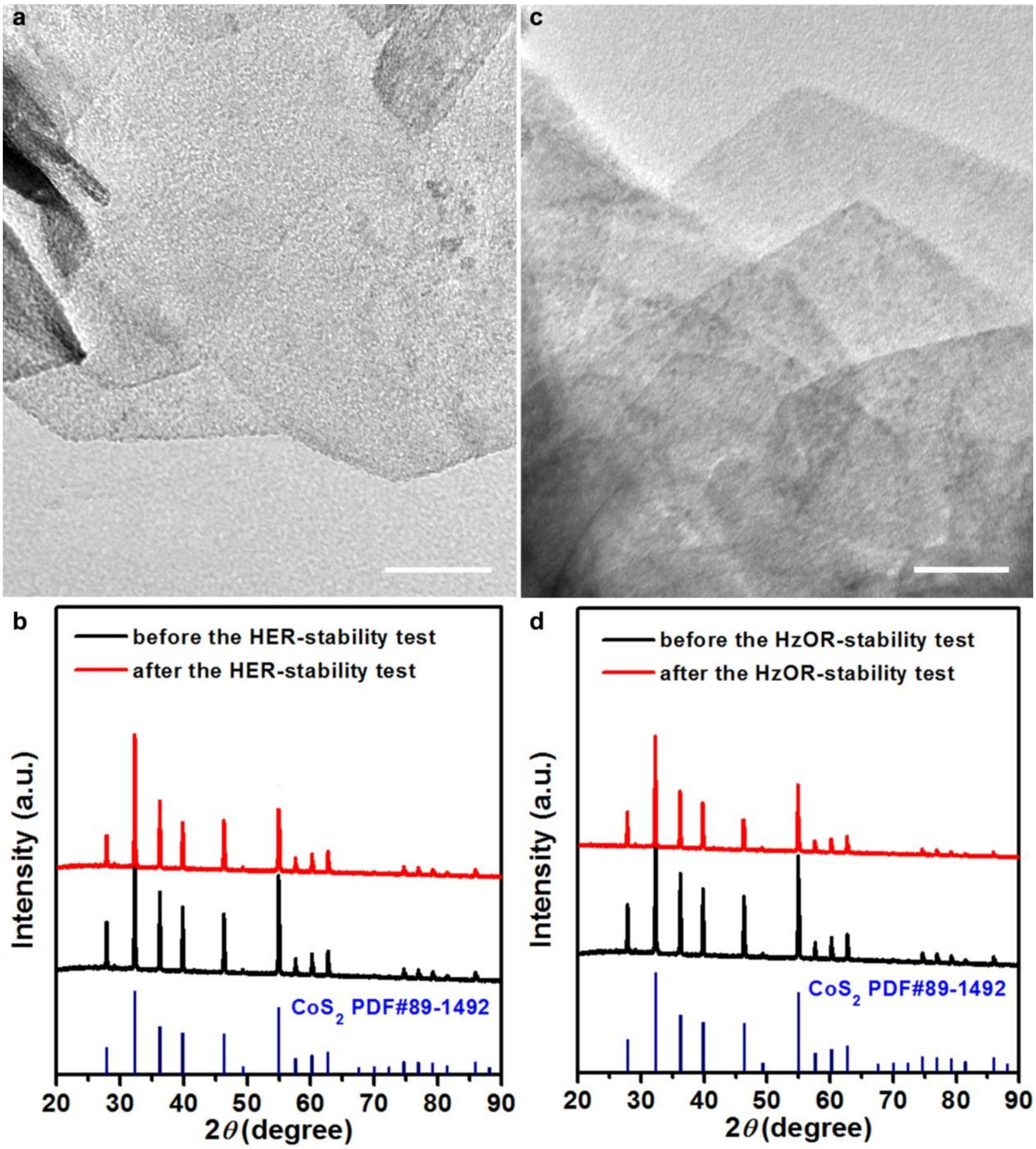

Supplementary Figure 13 | TEM images and XRD patterns of the Fe-CoS_2 nanosheets after the stability tests. a, **b** TEM image and XRD pattern after the HER-stability test. **c**, **d** TEM image and XRD pattern after the HzOR-stability test. Note that the black patterns in **b** and **d** are the same black one in Fig. 1a, the standard pattern of CoS_2 is added in blue in **b** and **d**, and they are redrawn here for comparison. Comparing these results and Fig. 1a, b shows that the Fe-CoS_2 nanosheets preserve the same structure and morphology before and after the

chronoamperometric experiments, indicating their structural stability. Scale bars in **a** and **c** are 50 nm.

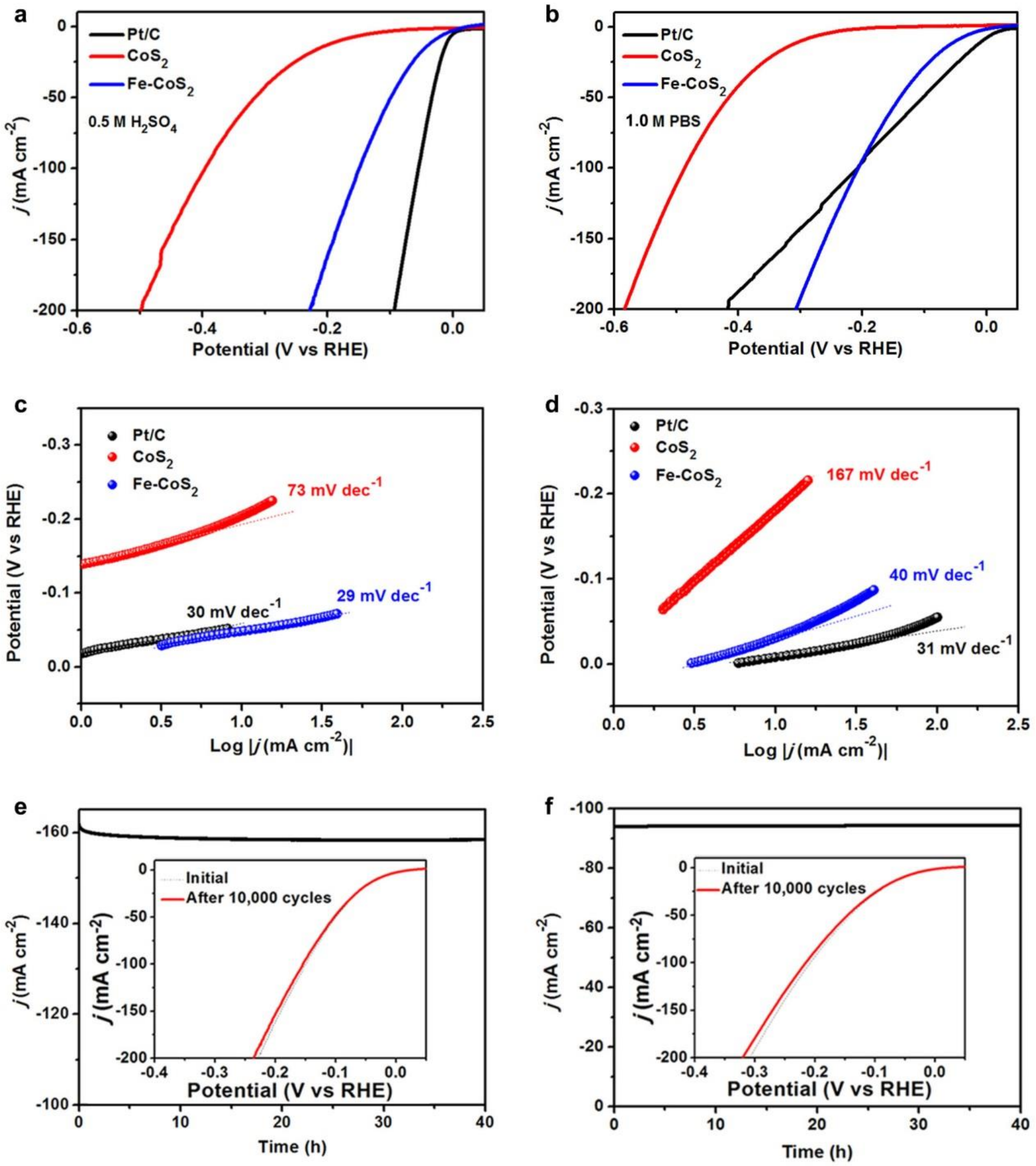

Supplementary Figure 14 | Electrochemical performances of the Fe-CoS_2 nanosheets for HER. **a**, **b** HER polarization curves of 20 wt.% Pt/C, the pure CoS_2 nanosheets and the Fe-CoS_2 ones in 0.5 M H_2SO_4 and 1.0 M PBS. **c**, **d** Tafel plots corresponding to **a**, **b**. **e**, **f** Chronoamperometric curves recorded on the Fe-CoS_2 nanosheets for 40 h at a constant working potential of – 0.2 V for HER in 0.5 M H_2SO_4 and 1.0 M PBS. The insets in **e** and **f** are the HER polarization curves of the Fe-CoS_2

nanosheets before and after 10,000 potential cycles in 0.5 M H_2SO_4 and 1.0 M PBS, respectively. It should be noted that in 1.0 M PBS, Pt/C exhibited a typical 0 mV overpotential at the onset position, and its corresponding HER current density started to increase from 0 earlier and faster than that of Fe-CoS_2; but, when the applied potential was increased further, the increase rate of the HER current density of Pt/C was lower than that of Fe-CoS_2, due to the larger charge transfer resistance of Pt/C (see Supplementary Fig. 10 for details); thus, after the applied potential was increased to be larger than a critical value (that is, the HER current density was larger than a critical value), the HER current density of Fe-CoS_2 started to be larger than that of Pt/C, as displayed in **b** here, showing better HER activities than Pt/C. In contrast, in 0.5 M H_2SO_4, the charge transfer resistance of Fe-CoS_2 is larger than that of Pt/C (see Supplementary Fig. 10 for details), and thus its HER activities were always worse than those of Pt/C, as displayed in **a** here.

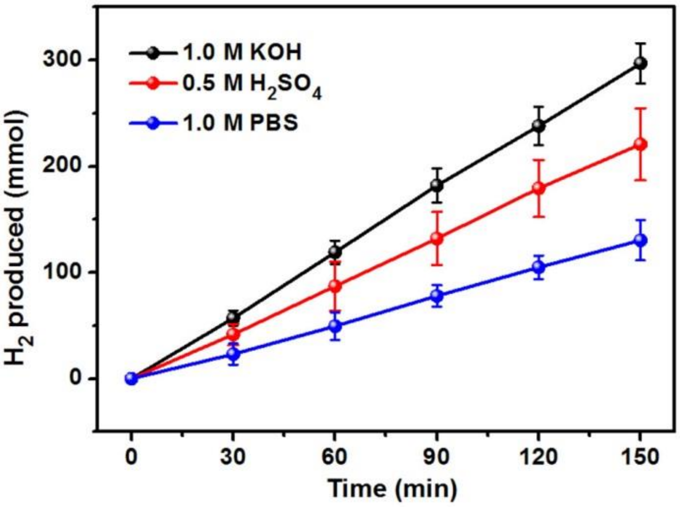

Supplementary Figure 15 | Actual H_2 yields catalyzed by the Fe-CoS_2 nanosheets at a constant j_{HER} of 50 mA cm^{-2}, and their linear fitting results. The error bars represent the standard deviations of the yields. The Faradaic efficiency (FE) of the Fe-CoS_2 nanosheets for the generation of H_2 during an HER process can be obtained from the H_2 yield using the formula FE (%) = $2n_{H2} / (j_{HER}t)$, where n_{H2} is the H_2 yield and t is the HER time[6–8].

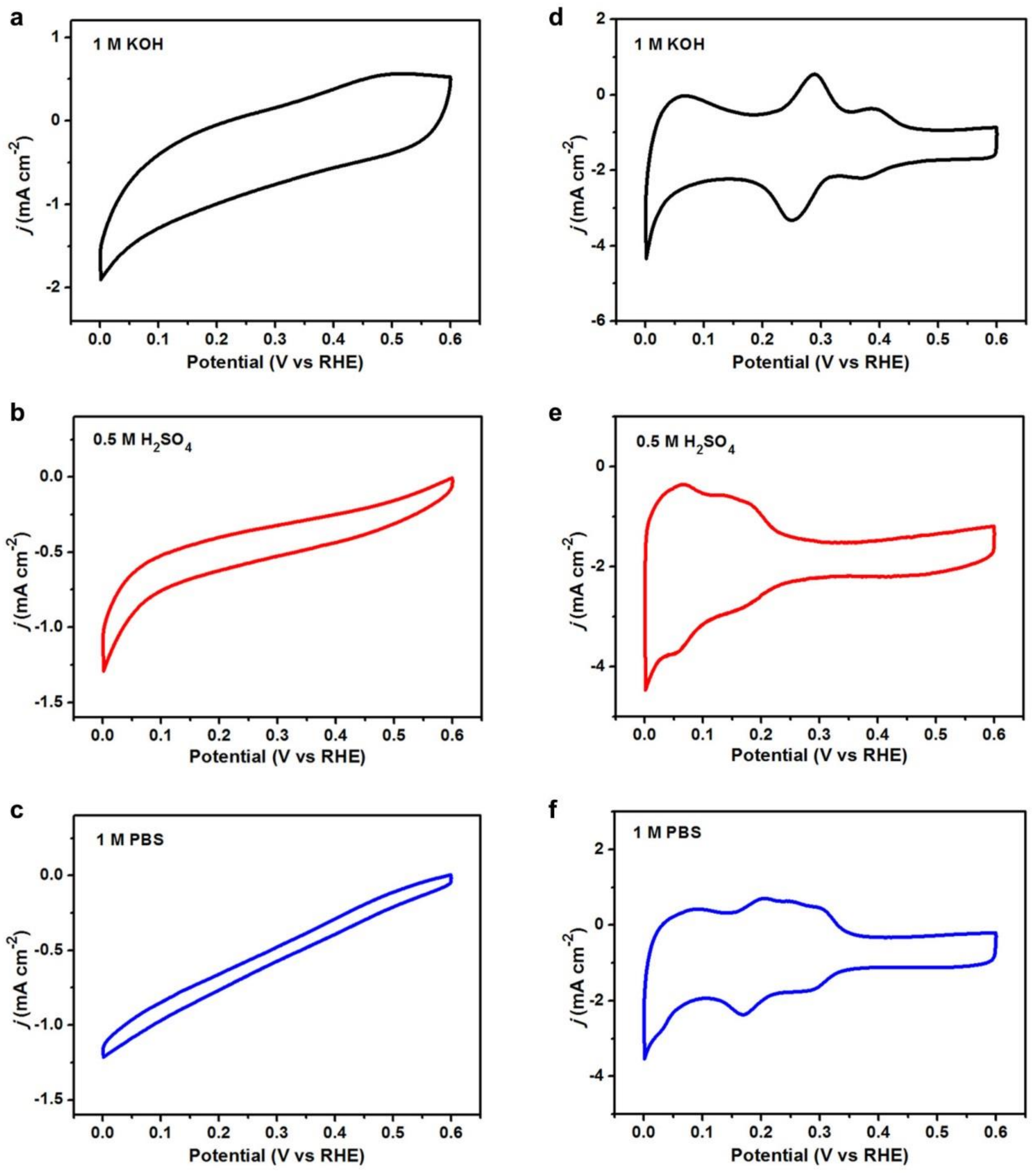

Supplementary Figure 16 | CV curves measured in different electrolytes. a–**c** CV curves of the Fe-CoS_2 nanosheets. **d**–**f** CV curves of Pt/C. By the curves in **a**–**c** here (see more details in Methods), the Q_s values of Fe-CoS_2 were measured to be 7.28 × 10^{-3} C cm^{-2} (1.0 M KOH), 5.32 × 10^{-3} C cm^{-2} (0.5 M H_2SO_4) and 6.44 × 10^{-3} C cm^{-2} (1.0 M PBS). Thus, the *n* values of Fe-CoS_2 were calculated to be 7.54 × 10^{-8} mol cm^{-2} (1.0 M KOH), 5.51 × 10^{-8} mol cm^{-2} (0.5 M H_2SO_4) and 6.67 × 10^{-8} mol cm^{-2} (1.0 M

PBS). Similarly, by the curves in **d**–**f** here, the Q_s values of Pt/C were measured to be 1.69×10^{-2} C cm^{-2} (1.0 M KOH), 1.94×10^{-2} C cm^{-2} (0.5 M H_2SO_4) and 1.20×10^{-2} C cm^{-2} (1.0 M PBS). Thus, the n values of Pt/C were calculated to be 1.75×10^{-7} mol cm^{-2} (1.0 M KOH), 2.01×10^{-7} mol cm^{-2} (0.5 M H_2SO_4) and 1.24×10^{-7} mol cm^{-2} (1.0 M PBS).

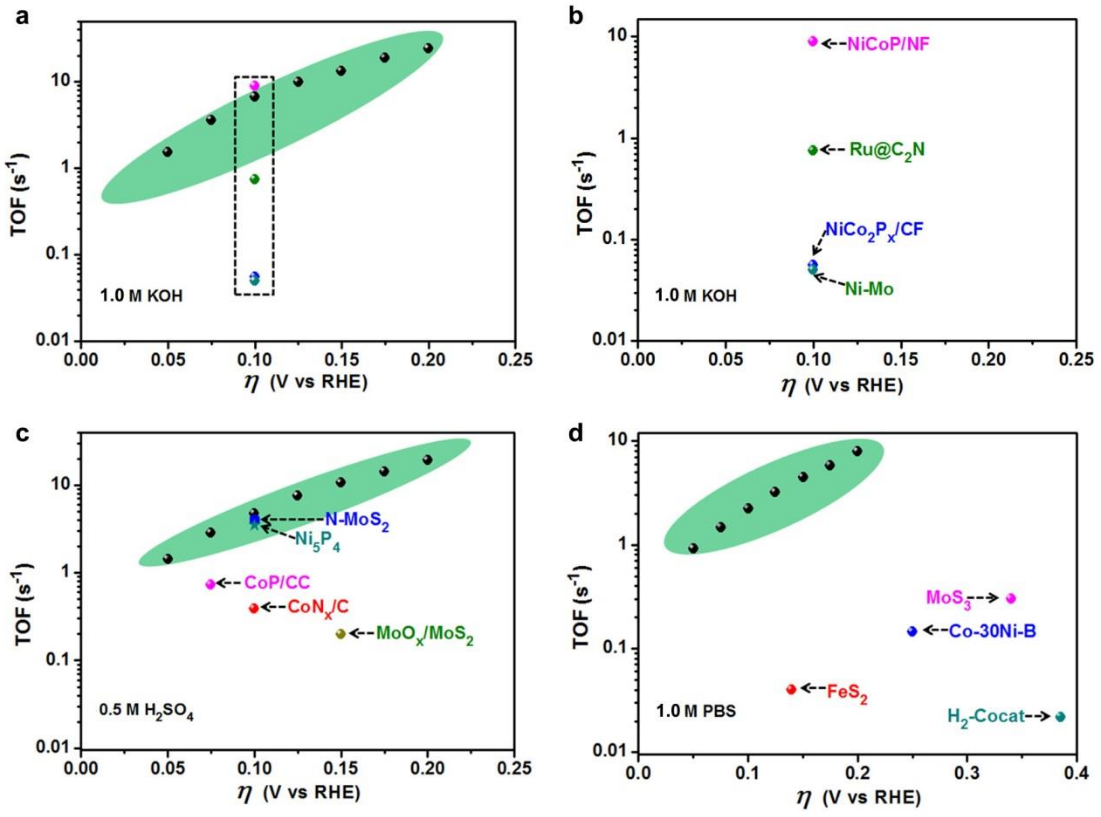

Supplementary Figure 17 | Comparison of the TOF values of different electrocatalysts for HER. a–d TOF values of the Fe-CoS_2 nanosheets (denoted by the black dots and highlighted by the light-green-coloured area) and other recent HER electrocatalysts (denoted by the color dots; their names are labeled) in 1.0 M KOH (**a**, **b**), 0.5 M H_2SO_4 (**c**) and 1.0 M PBS (**d**). **b** is an enlargement of the boxed area in **a**. Data adapted from: ref. 9 for NiCoP/NF; ref. 10 for Ru@C_2N; ref. 11 for $NiCo_2P_x$/CF; ref. 12 for Ni-Mo; ref. 13 for CoN_x/C; ref. 14 for N-MoS_2; ref. 15 for Ni_5P_4; ref. 16 for CoP/CC; ref. 17 for MoO_x/MoS_2; ref. 18 for FeS_2; ref. 19 for Co-30Ni-B; ref. 20 for H_2-Cocat.; ref. 21 for MoS_3.

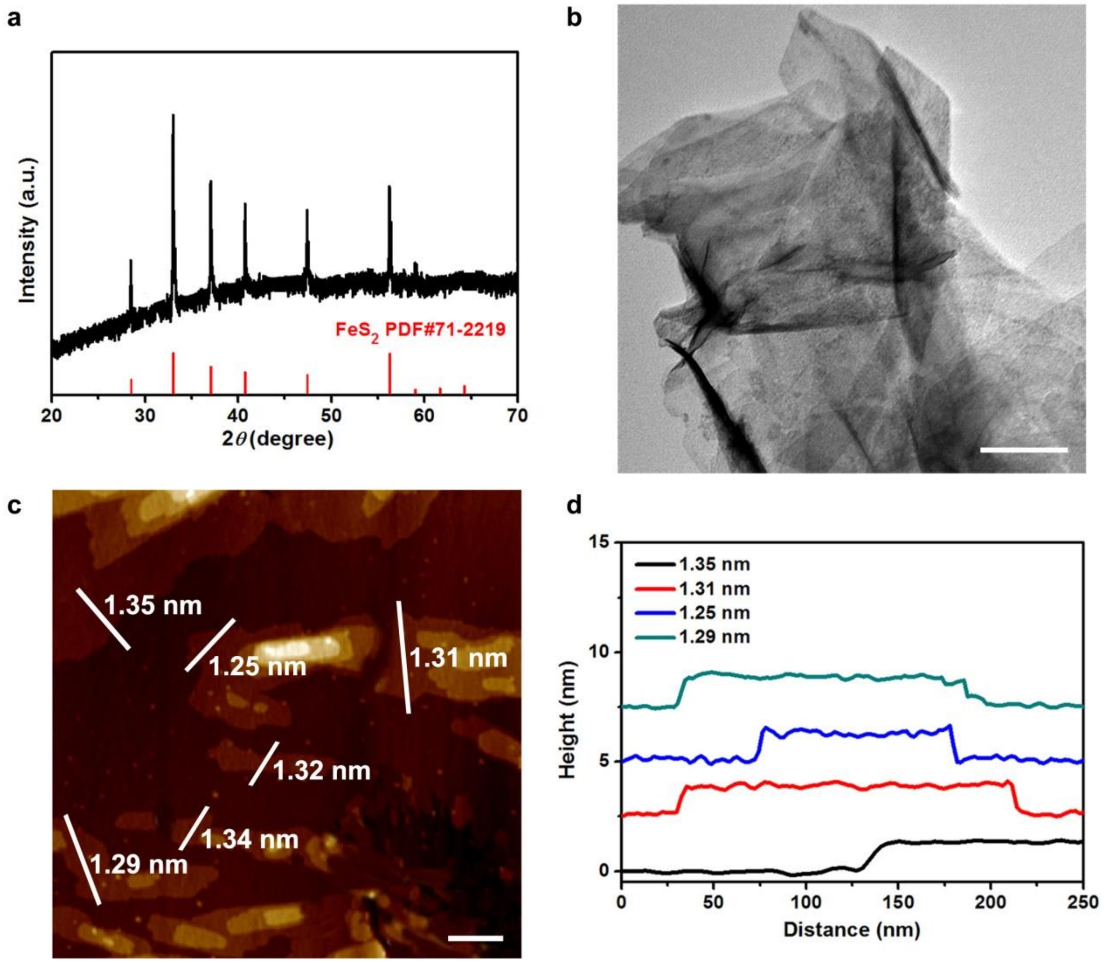

Supplementary Figure 18 | Characterization of the pure FeS_2 nanosheets. a XRD pattern, in which the standard pattern of FeS_2 is added in red for comparison. **b** TEM image. **c** AFM image, in which the six values are the thickness values measured by the height profiles along the corresponding white lines. **d** Height profiles along four white lines in **c**, which are randomly selected as examples. The six values in **c** give an average of 1.31 nm and a standard deviation of 0.04 nm. Scale bars in **b** and **c** are 100 nm.

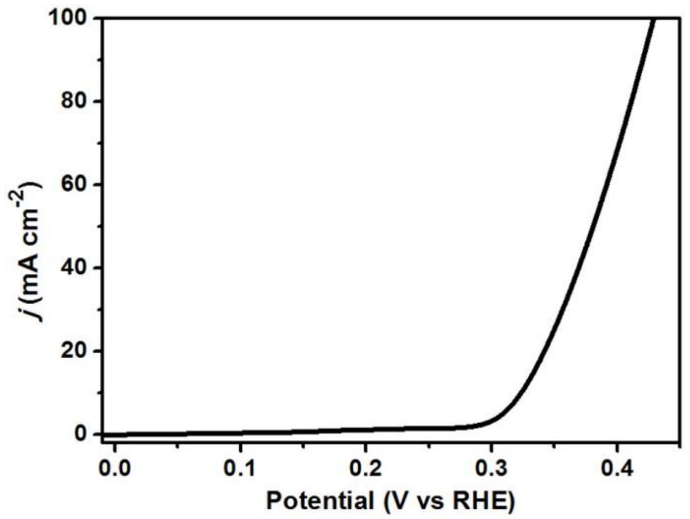

Supplementary Figure 19 | HzOR polarization curve of the pure FeS_2 nanosheets in 1.0 M KOH with 0.1 M hydrazine. The conditions to take this curve are the same as those for Fig. 2d. This curve shows that the FeS_2 nanosheets exhibited a potential of 428 mV for delivering the current density of 100 mA cm^{-2} (namely E_{100} = 428 mV). This E_{100} value is significantly larger than those of CoS_2 (205 mV) and Fe-CoS_2 (129 mV), as shown in Fig. 2d. This comparison indicates the inferior HzOR activity of FeS_2.

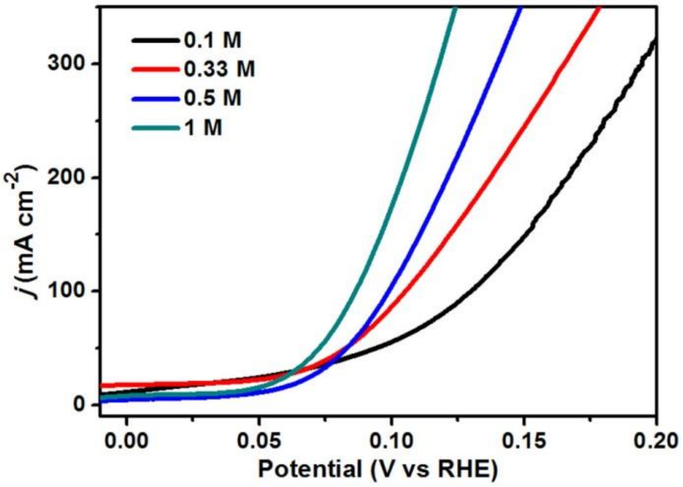

Supplementary Figure 20 | HzOR polarization curves of the Fe-CoS_2 nanosheets in hydrazine with different concentrations, whose values are labeled in the panel. Note that the blue curve in Fig. 2d is redrawn here as the black one. The conditions to measure the HzOR polarization curves here are the same as those for Fig. 2d. For instance, the KOH concentration in the solutions containing hydrazine was always kept at 1.0 M, the same as that for Fig. 2d.

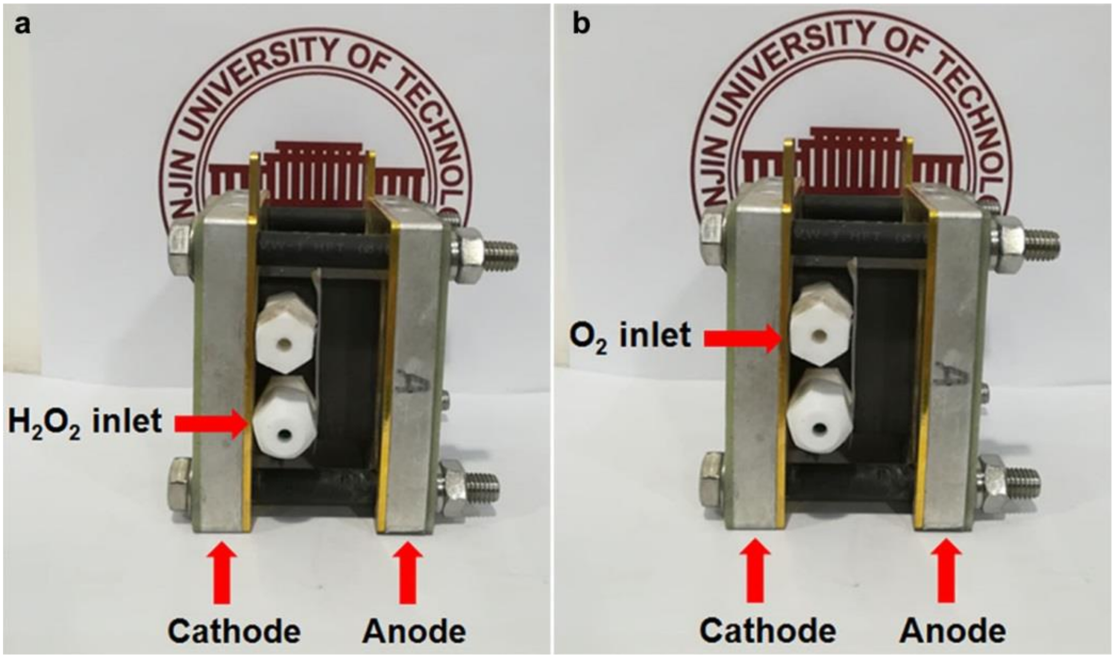

Supplementary Figure 21 | Optical images of the DHzFCs. a Optical image of the DHzFC with H_2O_2 as the oxidizing agent. **b** Optical image of the DHzFC with O_2 as the oxidizing agent.

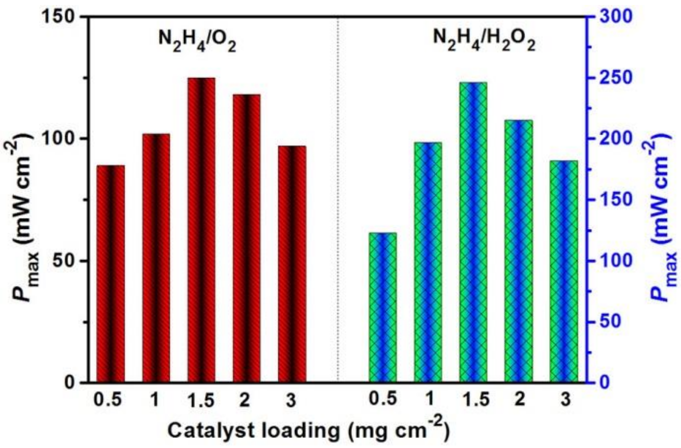

Supplementary Figure 22 | P_{max} of the DHzFCs, whose anode and cathode were the Fe-CoS_2 nanosheets and 40 wt.% Pt/C, respectively, as a function of Fe-CoS_2 loadings. P_{max} has the same meaning as that in the main text, which is the maximum power density. The results here indicate that the best Fe-CoS_2 loading corresponding to the highest P_{max} value is 1.5 mg cm^{-2} for the DHzFCs with O_2 or H_2O_2 as the oxidizing agent.

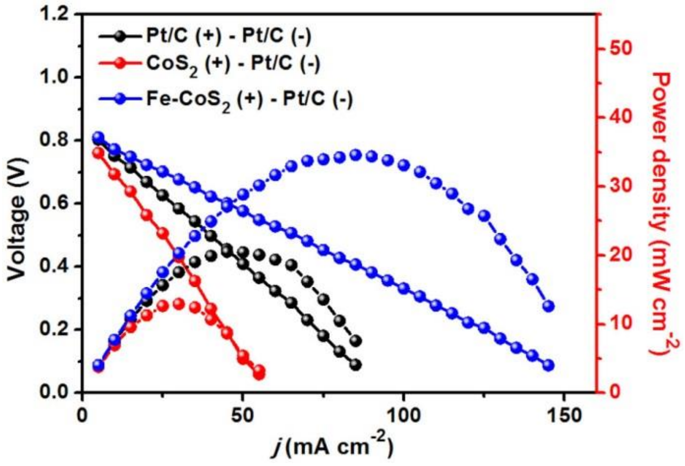

Supplementary Figure 23 | Current density–voltage (*j*-*V*) and current density–power density (*j*-*P*) curves of three DHzFCs. The three DHzFCs use the Fe-CoS_2 nanosheets, the CoS_2 nanosheets and 40 wt.% Pt/C as the anodes and 40 wt.% Pt/C as the cathodes, in which the oxidizing agents are all air.

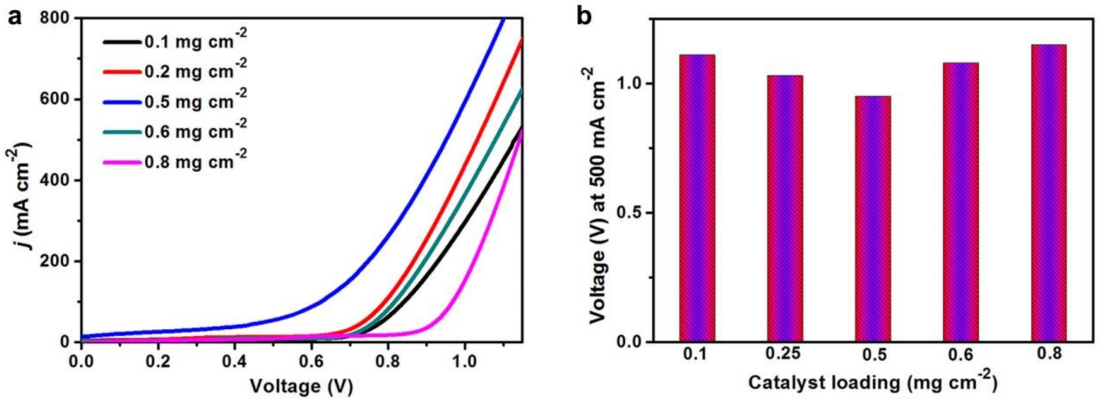

Supplementary Figure 24 | OHzS performances of the Fe-CoS_2 nanosheets with different loadings. **a** OHzS polarization curves at different loadings. **b** Required cell voltage for 500 mA cm^{-2}, corresponding to **a**. Note that the blue curve in Fig. 4a is redrawn here as the blue one in **a**. The results here indicate that the best Fe-CoS_2 loading corresponding to the highest OHzS performance (namely the lowest voltage for 500 mA cm^{-2}) is 0.5 mg cm^{-2}.

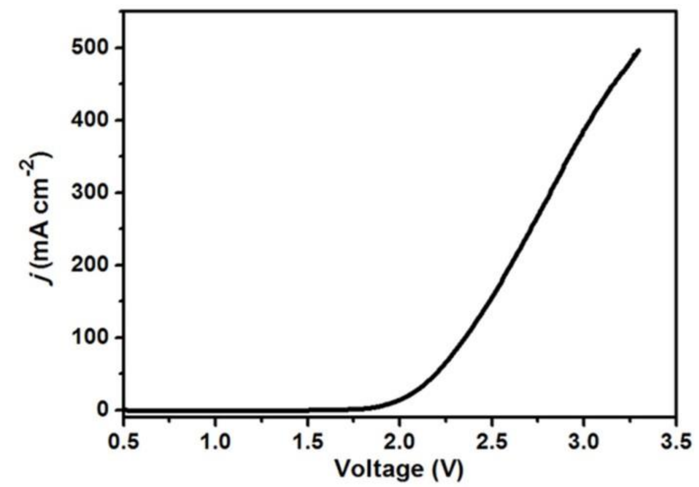

Supplementary Figure 25 | Polarization curve of the Fe-CoS_2 nanosheets for overall water splitting. During collecting the curve, the Fe-CoS_2 nanosheets worked bifunctionally for overall water splitting in 1.0 M KOH.

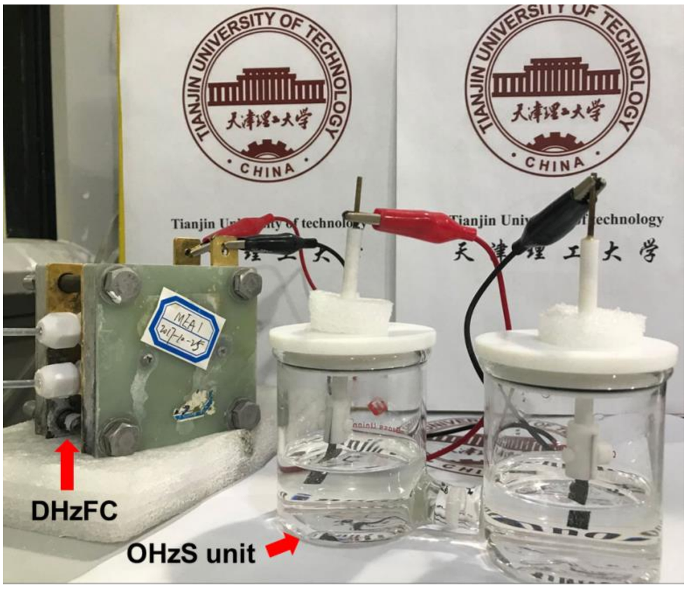

Supplementary Figure 26 | Optical image of a self-powered H_2 production system.

This self-powered H_2 production system integrates a DHzFC and an OHzS unit.

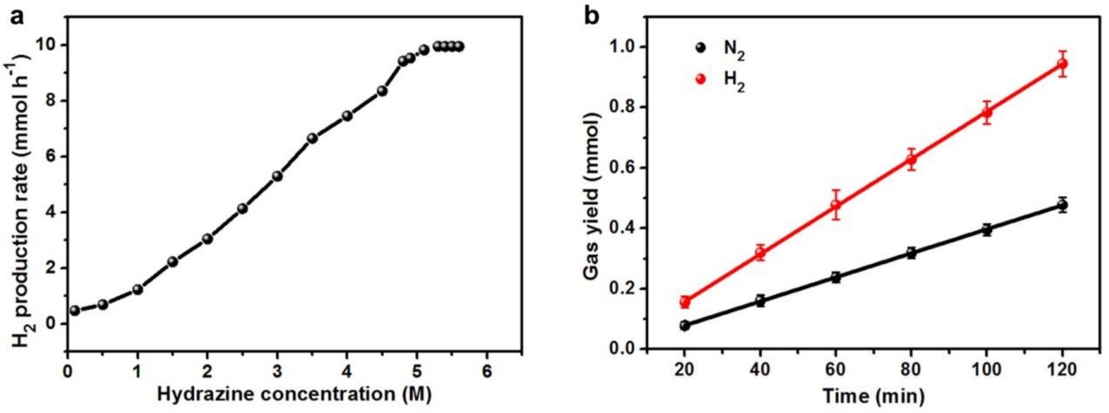

Supplementary Figure 27 | Performances of the self-powered H_2 production system with the Fe-CoS_2 nanosheets at different hydrazine concentrations at 0.7 V. **a** H_2 production rates at different hydrazine concentrations. **b** Generated amounts of H_2 and N_2 in the system with 0.1 M hydrazine, in which the error bars represent the standard deviations of the amounts. The linear relationship in **b** gives the H_2 production rate to be 0.47 mmol h^{-1}. The result in **a** shows that when the hydrazine concentration increased from 0.1 to 5.6 M, the H_2 production rate exhibited an initial value of 0.47 mmol h^{-1}, then increased and finally became saturated at 5.3–5.6 M. The saturated value is 9.95 mmol h^{-1}, corresponding to Fig. 4d.

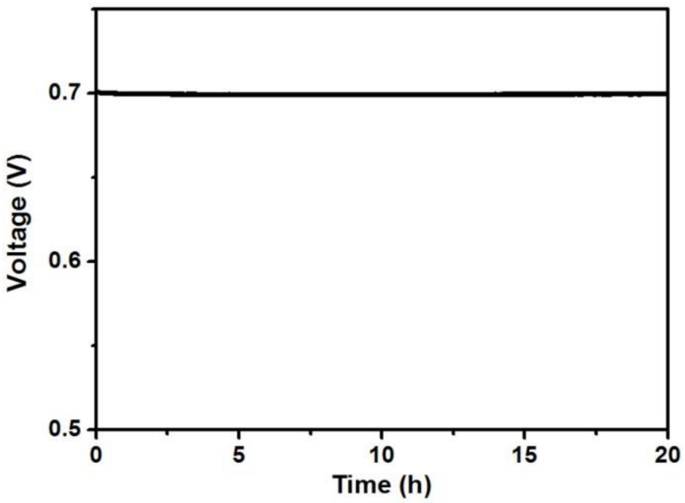

Supplementary Figure 28 | Voltage–time (*V*–*t*) curve of the self-powered H_2 production system. This self-powered H_2 production system integrates a DHzFC and an OHzS unit.

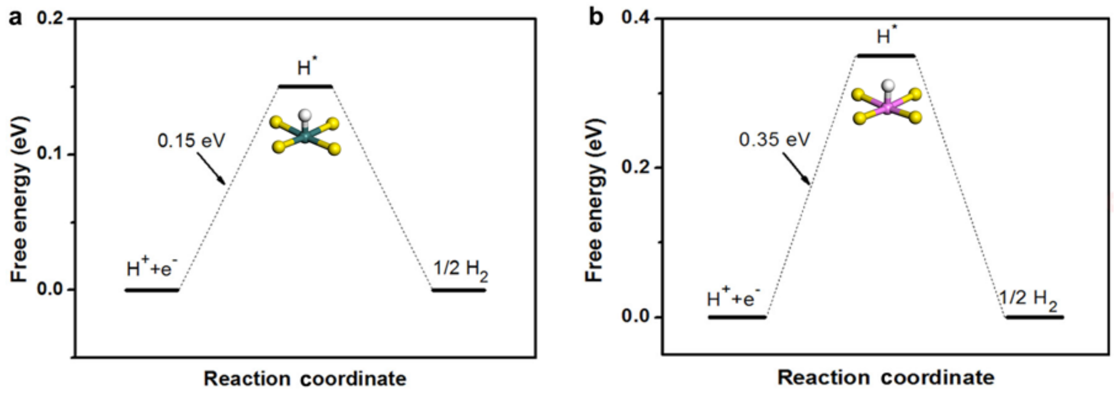

Supplementary Figure 29 | Calculated free-energy diagrams of HER at 0 V vs RHE. a Free-energy diagram of HER on the Co site of the Fe-CoS_2 surface. **b** Free-energy diagram of HER on the Fe site of the Fe-CoS_2 surface. The insets in **a** and **b** are the atomic configurations of H^* adsorbed on the Co and the Fe sites, respectively, of the Fe-CoS_2 surface. The ΔG_{H*} value on the Co site was calculated to be +0.15 eV, smaller than that on the Fe site (+0.35 eV), suggesting that the atomic configuration of H^* adsorbed on the Co site is more stable than on the Fe site.

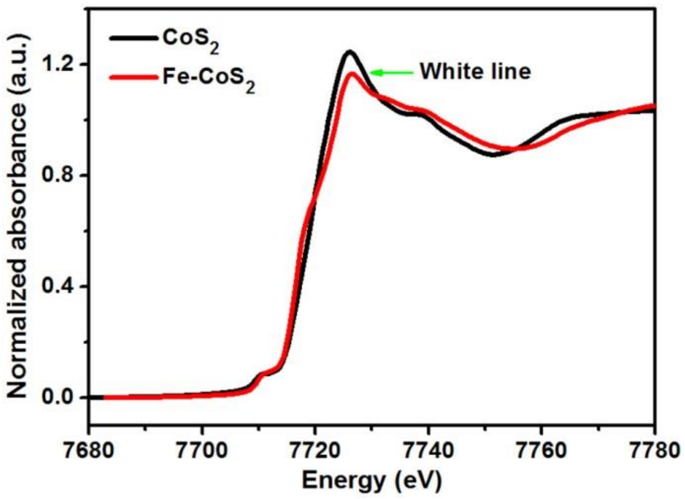

Supplementary Figure 30 | Co K-edge XANES spectra of the pure CoS_2 nanosheets and the Fe-CoS_2 ones. The white line means the first peak after absorption edge.

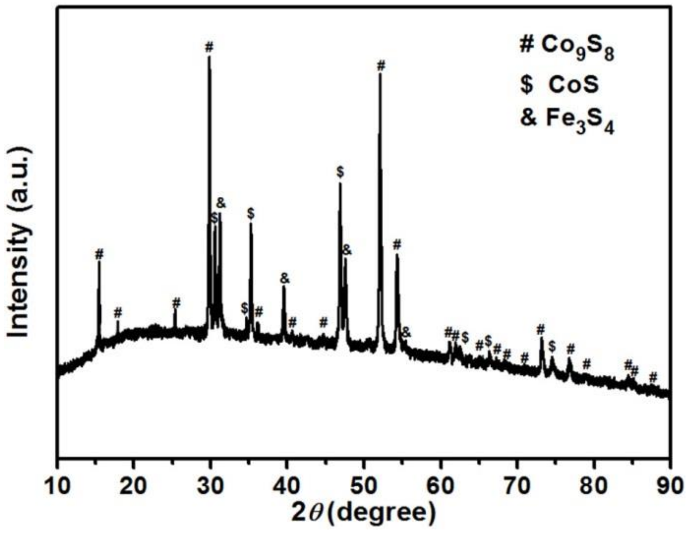

Supplementary Figure 31 | XRD pattern of the Fe-CoS$_2$ nanosheets. These Fe-CoS$_2$ nanosheets have the Fe doping content of 9.5 at.%.

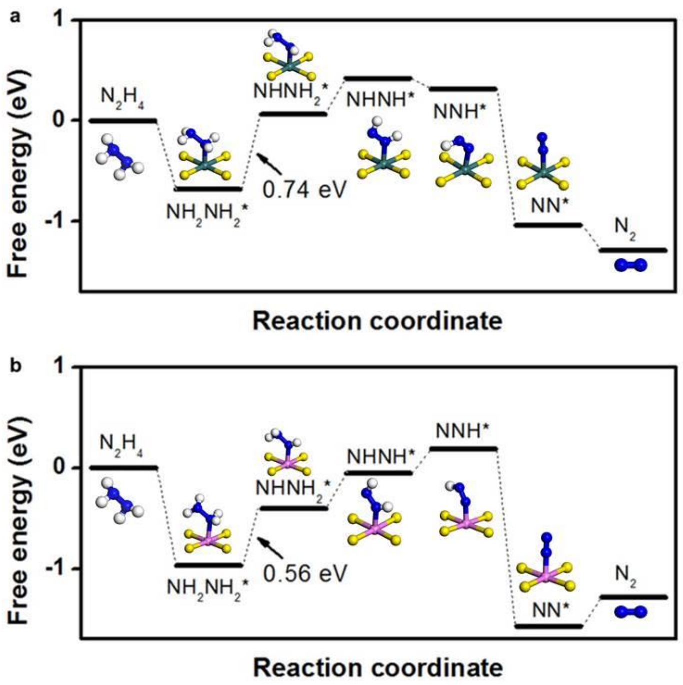

Supplementary Figure 32 | Calculated free-energy diagrams of HzOR at 0 V vs RHE. **a** Free-energy diagram of HzOR on the Co site of the Fe-CoS_2 surface. **b** Free-energy diagram of HzOR on the Fe site of the Fe-CoS_2 surface. The insets in **a** and **b** are the N_2H_4 and N_2 molecular models and the atomic configurations of the intermediates adsorbed on the Co and the Fe sites, respectively, of the Fe-CoS_2 surface. The ΔG value of PDS on the Co site was calculated to be +0.74 eV, larger than that on the Fe site (+0.56 eV), suggesting that the atomic configurations of intermediates on the Fe site are more stable than on the Co site.

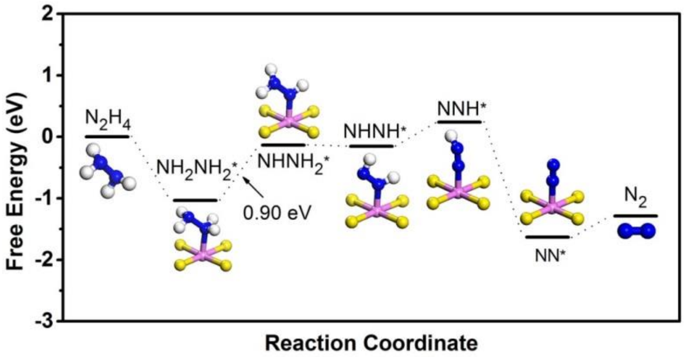

Supplementary Figure 33 | Free energy diagram of HzOR on the FeS_2 surface at 0 V vs RHE. The insets are the N_2H_4 and N_2 molecular models and the most stable configurations of the intermediates adsorbed on the FeS_2 surface.

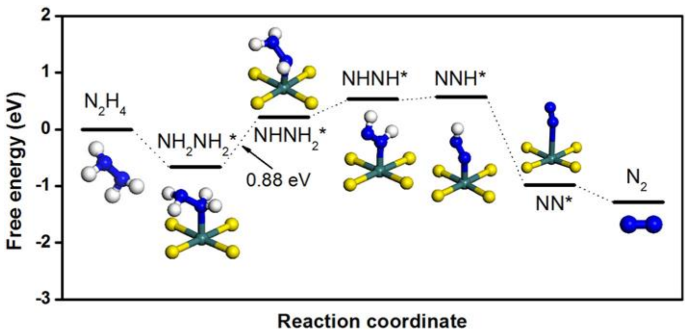

Supplementary Figure 34 | Free energy diagram of HzOR on the CoS_2 surface at 0 V vs RHE. The insets are the N_2H_4 and N_2 molecular models and the most stable configurations of the intermediates adsorbed on the CoS_2 surface.

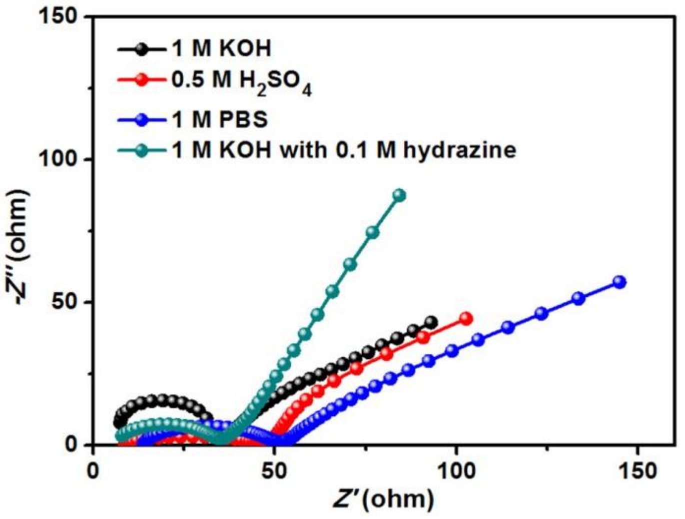

Supplementary Figure 35 | EIS plots recorded in different electrolytes by using the Fe-CoS$_2$ nanosheets at an open-circuit potential. The EIS spectra give the solution resistances to be 7.4 Ω (1.0 M KOH), 9.0 Ω (0.5 M H_2SO_4), 13.9 Ω (1.0 M PBS) and 7.9 Ω (1.0 M KOH with 0.1 M hydrazine), respectively.

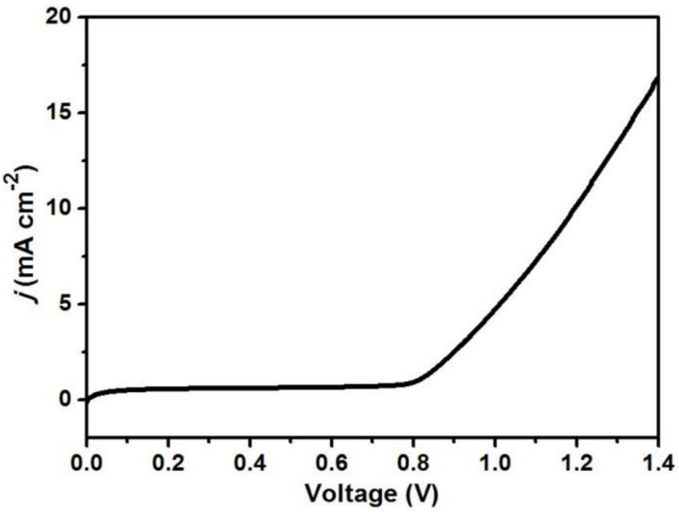

Supplementary Figure 36 | OHzS polarization curve of the Ni foams in 1.0 M KOH with 0.1 M hydrazine. This curve shows that the Ni foams required a cell voltage of 1.20 V to achieve the OHzS current density of 10 mA cm^{-2}. This cell voltage is much higher than that of Fe-CoS_2 (0.002 V) for 10 mA cm^{-2}, which is shown in Fig. 4a. Moreover, when the cell voltage was 0.002 V, the current density of the Ni foams was – 0.01 mA cm^{-2}, far lower than that of Fe-CoS_2 (10 mA cm^{-2}). That is, if we use the 10 mA cm^{-2} of Fe-CoS_2 as a reference, the – 0.01 mA cm^{-2} of the Ni foams is very close to zero. Besides, the negative sign of the – 0.01 mA cm^{-2} indicates that the OHzS did not occur at that moment. Therefore, Ni foams gave a negligible contribution to the observed OHzS activity of Fe-CoS_2 on Ni foams.

Supplementary Table 1 | HER catalytic performances in our work and the literature.

Catalyst	Electrolyte	η_{10} (mV)	Durability	Reference
**MoC_x**	0.5 M H_2SO_4	142	11 h	*Nat. Commun.* **2015,** *6*, 6512
	1.0 M KOH	151	11 h	
**Mo_xC-Ni@NCV**	0.5 M H_2SO_4	68	50,000 s	*J. Am. Chem. Soc.* **2015,** *137*, 15753
	1.0 M KOH	126	10,000 s	
**CoP**	0.5 M H_2SO_4	65	5,000 cycles	*J. Am. Chem. Soc.* **2014,** *136*, 7587
	1.0 M PBS	106	1,000 cycles	
	1.0 M KOH	209	1,000 cycles	
**CoS_2@NSC/CFP**	0.5 M H_2SO_4	95	1,000 cycles	*ChemCatChem* **2017**, 10, 796

**MoS_2/CoS_2**	0.5 M H_2SO_4	87	1,000 cycles	*J. Mater. Chem. A* **2015,** *3,* 22886
**CoS_2/P**	0.5 M H_2SO_4	67	3,000 cycles	*Chem. Commun.* **2015,** *51,* 14160
**CoS_2**	0.5 M H_2SO_4	107	3,000 cycles	*Green Energy Environ.* **2017,** *2,* 134
**CoS_2/RGO-CNT**	0.5 M H_2SO_4	142	500 cycles	*Angew. Chem.* **2014,** *126,* 12802
**CoS_2 NW**	0.5 M H_2SO_4	145	41 h	*J. Am. Chem. Soc.* **2014,** *136,* 10053
**N_s-V_s-CoS_2**	0.5 M H_2SO_4	57	10,000 cycles	*ACS Energy Lett.* **2017,** *2,* 1022
**CoS_2 pyramids**	1.0 M KOH	244	30,000 s	*Electrochimica Acta* **2014,** *148,* 170
**CoS_2 NA/Ti**	1.0 M KOH + 0.3 M Urea	140	15,000 cycles	*Electrochimica Acta* **2017,** *246,* 776
**$Ni(OH)_2$-CoS_2**	1.0 M KOH	99	30 h	*Nanoscale* **2017,** *9,* 16632
**Ni_5P_4-Ni_2P-NS**	0.5 M H_2SO_4	120	72 h	*Angew. Chem. Int. Ed.* **2015,** *54*, 8188
**NiSe/NF**	1.0 M KOH	96	12 h	*Angew. Chem. Int. Ed.* **2015,** *54*, 9351

**Ni_3S_2/NF**	neutral media	170	200 h	*J. Am. Chem. Soc.* **2015,** *137*, 14023
**p-1T-MoS_2**	0.5 M H_2SO_4	153	20,000 s	*J. Am. Chem. Soc.* **2016,** *138*, 7965
**α-iron-nickel sulfide**	0.5 M H_2SO_4	105	40 h	*J. Am. Chem. Soc.* **2015,** *137,* 11900
**CoMoP@C**	0.5 M H_2SO_4	41	10,000 cycles	*Energy Environ. Sci.* **2017,** *10*, 788
	alkaline electrolyte	81		
	neutral electrolyte	526 @ 50 mA cm^{-2}	24 h	
**$NiCo_2P_x$/CF**	0.5 M H_2SO_4	104	5,000 cycles	*Adv. Mater.* **2017,** *29*, 1605502
	1.0 M KOH	58		
	1.0 M PBS	63	30 h	
**CoP@BCN-1**	0.5 M H_2SO_4	87	2,000 cycles	*Adv. Energy Mater.* **2017,** *7,* 1601671
	1.0 M KOH	215		
	1.0 M PBS	122		

**Co/CoP$_x$**	0.5 M H_2SO_4	178	12 h	*Adv. Energy Mater.* **2017,** *7,* 1602355
	1.0 M KOH	253		
	1.0 M PBS	138		
**HNDCM-Co/CoP**	0.5 M H_2SO_4	135	20 h	*ACS Nano* **2017,** *11*, 4358
	1.0 M KOH	138		
**Ru@C$_2$N**	0.5 M H_2SO_4	22	10,000 cycles	*Nat. Nanotech.* **2017,** *12*, 441
	1.0 M KOH	17		
**Ni$_5$P$_4$ pellet**	1.0 M H_2SO_4	23	16 h	*Energy Environ. Sci.* **2015,** *8*, 1027
	1.0 M NaOH	49		
**ONPPGC/OCC**	0.5 M H_2SO_4	386	10 h	*Energy Environ. Sci.* **2016,** *9*, 1210
	1.0 M KOH	446		
	0.2 M PBS	352 @ 1 mA cm^{-2}		

**Ni-C-N NSs**	0.5 M H_2SO_4	60.9	70 h	*J. Am. Chem. Soc.* **2016,** *138*, 14546
	1.0 M KOH	30.8	70 h	
	1.0 M PBS	92.1	70 h	
**Pt/C**	0.5 M H_2SO_4	11	-	*This work*
	1.0 M KOH	23		
	1.0 M PBS	44		
**Fe-CoS$_2$ nanosheets**	0.5 M H_2SO_4	31	10,000 cycles<br>40 h	
	1.0 M KOH	40		
	1.0 M PBS	49		

η_{10} is η at 10 mA cm^{-2} except those specified else.

Supplementary Table 2 | TOF values in the HER performances of our work and the literature.

Catalyst	Electrolyte	TOF (s^{-1}) at η = 100 mV	TOF (s^{-1}) at η = 200 mV	Reference
**Ni-C-N NSs**	0.5 M H_2SO_4	0.44	6.67	*J. Am. Chem. Soc.* **2016,** *138*, 14546
	1.0 M PBS	0.29	0.95	
	1.0 M KOH	1.95	8.52	
**CoNx/C**	0.5 M H_2SO_4	0.39	6.5	*Nat. Commun.* **2015,** *6*, 7992
**Ni_2P**	0.5 M H_2SO_4	0.015	0.5	*J. Am. Chem. Soc.* **2013,** *135*, 9267
**CoP**	0.5 M H_2SO_4	0.046	-	*Angew. Chem. Int. Ed.* **2014,** *53*, 5427
**Ni-Mo**	2.0 M KOH	0.05	0.36	*ACS Catal.* **2013,** *3*, 166
**FeP/Ti**	0.5 M H_2SO_4	0.277	-	*ACS Nano* **2014,** *8*, 11101
**FeS_2**	0.1 M PBS	0.04 (140 mV)	-	*ACS Catal.* **2015,** *5*, 6653

**N-MoS_2-3**	0.5 M H_2SO_4	4	-	*Adv. Energy Mater.* **2017,** *7*, 1602086
**Ni-MoS_2**	0.5 M H_2SO_4	30.9 (650 mV)	60.3 (740 mV)	*ACS Catal.* **2016,** *6,* 6008
**p-1T-MoS_2**	0.5 M H_2SO_4	0.5 (153 mV)	-	*J. Am. Chem. Soc.* **2016,** *138*, 7965
**Irradiated Au-MoS_2**	0.5 M H_2SO_4	8.76 (300 mV)	-	*J. Am. Chem. Soc.* **2015,** *137*, 7365
**CoS**	0.5 M H_2SO_4	0.39	-	*J. Mater. Chem. A* **2015,** *3,* 13066
**MoS_2**	0.5 M H_2SO_4	-	0.725 (300 mV)	*Adv. Mater.* **2013,** *25,* 5807
**NiCoP/rGO**	0.5 M H_2SO_4	1.70	-	*Adv. Funct. Mater.* **2016,** *26,* 6785
**NiCoP/NF**	1.0 M KOH	8.93	3.88 (300 mV)	*Nano Lett.* **2016,** *16,* 7718
**Ni_2P/Ni/NF**	1.0 M KOH	-	0.015 (350 mV)	*ACS Catal.* **2016,** *6,* 714
**1D-RuO_2-CN_x**	0.5 M H_2SO_4	-	0.0961 (350 mV)	*ACS Appl. Mater. Interfaces* **2016,** *8,* 28678
**$NiWS_x$**	neutral solution	-	0.12 (275 mV)	*Energy Environ Sci.* **2013,** *6,* 2452

**Ni-MoS_2**	1 M KOH	-	0.32 (150 mV)	*Energy Environ Sci.* **2016,** *9,* 2789
**Ni_5P_4 pellet**	1.0 M H_2SO_4	3.5	9.8	*Energy Environ. Sci.* **2015**, *8,* 1027
	1.0 M KOH	0.79	2.9	
**$NiCo_2P_x$/CF**	0.5 M H_2SO_4	0.021	-	*Adv. Mater.* **2017**, *29,* 1605502
	1.0 M KOH	0.056		
	1.0 M PBS	0.055		
**CoS_2 nanosheets**	0.5 M H_2SO_4	0.26 (400 mV)	1.34 (500 mV)	*This work*
	1.0 M KOH	0.23 (400 mV)	1.12 (500 mV)	
	1.0 M PBS	0.16 (400 mV)	0.69 (500 mV)	
**Fe-CoS_2 nanosheets**	0.5 M H_2SO_4	4.76	15.14	
	1.0 M KOH	6.74	20.35	
	1.0 M PBS	2.21	7.34	

Supplementary Table 3 | HzOR catalytic performances in our work and the literature.

Catalyst	Electrolyte	E_{100} (V vs RHE)	Durability	Reference
**FePc**	0.2 M KOH + (-) N2H4	~ 0.35	5,000 cycles	*Talanta* **2005,** *67*, 162
**NiHCF**	0.1 M $NaNO_3$ + (-) N_2H_4	0.5	100 cycles	*J. Electroanal. Chem.* **2008,** *617*, 111
**PEDOP**	3.0 M NaCl + (-) N_2H_4	- [a]	240,000 s	*Sens. Actuators B: Chem.* **2011,** *153*, 246
**Pd/CNTs**	0.01 M $N_2H_4SO_4$ + 0.1 M K_2SO_4 + (-) N_2H_4	~ 0.1	1,800 s	*Electrochem. Commun.* **2009,** *11*, 504
**PANI-Ag**	0.5 M HCl + 5×10^{-4} M N_2H_4	~ 1	-	*Colloid. Surf. A* **2011,** *377*, 28
**Cu nanoparticles**	0.1 M KOH + 0.01 M N_2H_4	~ 0.5	100 cycles	*J. Mater. Chem. A* **2014,** *2*, 4580
**SiAl/SiPy/FeTsP**	0.5 M KCl + 2×10^{-3} M N_2H_4	~ 0.4	100 cycles	*Electroanalysis* **2005,** *17,* 9

**CoHCF(Au)**	0.1 M KNO_3 + 2.5×10^{-4} M N_2H_4	~ 0.7	-	*Electrochim. Acta* **2014,** *139*, 88
**CoHCF**	0.5 M NaCl + 5×10^{-4} M N_2H_4	-	15 cycles	*J. Solid State. Electrochem.* **1998,** *2*, 30
**PPy/LS**	PBS + 5×10^{-4} M N_2H_4	-	1,200 s	*J. Electroanal. Chem.* **2016,** *9*, 3143
**n-Ag/POT**	0.1 M NaOH + 0.02 M N_2H_4	-	400,000 s	*J. Solid State. Electrochem.* **2015,** *19*, 2235
**3D PNNF**	3.0 M KOH + 0.5 M N_2H_4	~ 0.125	12,000 s	*Nano. Res.* **2015,** *8*, 3365
**Pd-NWNWs**	0.1 M $HClO_4$ + (-) N_2H_4	~ 0.35	6,000 s	*Electrochim. Acta* **2015,** *176*, 125
**FeN_4**	0.2 M NaOH + (-) N_2H_4	~ 0.4	-	*Electrochem. Commun.* **2013,** *30*, 34
**AuCu NPs**	0.1 M PBS + 3×10^{-4} M N_2H_4	~ 0.2	-	*Electroanlysis* **2012,** *24,* 2380
**Ru(HCF)**	Britton–Robinson aqueous buffer solution (pH=1.8) + 10^{-3} M N_2H_4	~ 0.96	-	*J. Appl. Electrochem.* **2010,** *40*, 375

**Ni_2P/NF**	1.0 M KOH + 0.5 M N_2H_4	– 0.025 (50 mA cm^{-2})	10 h	*Angew. Chem. Int. Ed.* **2017,** *56*, 842
**$Ni_{0.6}Co_{0.4}$-ANSA**	3.0 M KOH + 0.5 M N_2H_4	~– 1 (V vs SCE)	10,000 s	*Adv. Sci.* **2017,** *4,* 1600179
**Cu film**	3.0 M KOH + 1.0 M N_2H_4	~– 0.65 (V vs SCE)	5,000 s	*Adv. Mater.* **2015,** *27,* 2361
**Pt (111)**	0.1 M $HClO_4$ + 0.001 M NaCl + 0.01 M N_2H_4	~ 0.45	-	*ChemElectroChem* **2017,** *4*, 1130
**Pt/C**	1.0 M KOH + 0.1 M N_2H_4	0.170	-	*This work*
**CoS_2 nanosheets**		0.205	-	
**Fe-CoS_2 nanosheets**		0.129	40 h	

E_{100} is E at 100 mA cm^{-2} except those specified else.

[a] All of "-" mean that no values were reported for the corresponding parameters in the corresponding references.

Supplementary Table 4 | DHzFC performances in our work and the literature.

Catalyst	Anodic fuel	P_{max} (mW cm^{-2})	OCV (V)	Reference
**Vap-PM-CNF**	4.0 M KOH + 4.0 M N_2H_4	127.5 (60 °C)	~ 0.92 (N_2H_4/O_2)	*Angew. Chem. Int. Ed.* **2017,** *56*, 13513
**Co**	1.0 M KOH + 0.67 M N_2H_4	-	~0.97 (N_2H_4/O_2)	*Angew. Chem. Int. Ed.* **2007,** *46*, 8024
**$Ni_{0.6}Co_{0.4}$-ANSA**	4.0 M KOH + 20 wt.% N_2H_4	107.1 (85 °C)	1.78 (N_2H_4/H_2O_2)	*Adv. Sci.* **2017,** *4,* 1600179
**Co@Au/C**	2.0 M NaOH + 2.0 M N_2H_4	122.8 (60 °C)	1.79 (N_2H_4/H_2O_2)	*Int. J. Hydrogen Energy* **2017,** *42,* 15623
**Pd-Ni/C**	1.0 M KOH + 2.0 M N_2H_4	~160 (60 °C)	- (N_2H_4/O_2)	*J. Power. Sources* **2011,** *196,* 956
**CoN_x/C**	1.0 M NaOH + 4.0 M N_2H_4	110 (70 °C)	~0.94 (N_2H_4/O_2)	*J. Mater. Chem.* **2010,** *20,* 8139

**Zr-Ni alloy**	4.0 M NaOH + 2.0 M N_2H_4	84 (25 °C)	~0.9 (N_2H_4/O_2)	*J. Power. Sources* **2008,** *182,* 520
**NPGLs**	4.0 M NaOH + 10 wt.% N_2H_4	195 (80 °C)	1.20 (N_2H_4/H_2O_2)	*Sci. Rep.* **2012,** *2* ,941
**Cu film**	4.0 M KOH + 20 % N_2H_4	160.8 (80 °C)	~ 1.0 (N_2H_4/O_2)	*Adv. Mater.* **2015,** *27,* 2361
**Co-PPy/C**	1.0 M KOH + 5 wt.% N_2H_4	75 (50 °C)	0.73 (N_2H_4/air)	*J. Electrochemical. Soc.* **2014**, *161,* F889
**Fe-CoS_2 nanosheets**	4.0 M KOH + 20 wt.% N_2H_4	246 (80 °C)	1.80 (N_2H_4/H_2O_2)	*This work*
		125 (80 °C)	1.03 (N_2H_4/O_2)	

Supplementary Table 5 | OHzS performances in our work and the literature

Bifunctional catalysts	Electrolyte	Cell Voltage	Reference
**CoS_2/TiM**	1.0 M KOH + 0.1 M N_2H_4	0.81 V at *j* = 100 mA cm^{-2}	*New J. Chem.* **2017,** *41,* 4754
**Ni_2P/NF**	1.0 M KOH + 0.5 M N_2H_4	~ 0.45 V at *j* = 100 mA cm^{-2}	*Angew. Chem. Int. Ed.* **2017,** *56,* 842
**CoP/TiM**	1.0 M KOH + 0.1 M N_2H_4	0.2 V at *j* = 10 mA cm^{-2}	*ChemElectroChem* **2017,** *4,* 481
**FeP NA/NF**	1.0 M KOH + 0.5 M N_2H_4	0.5 V at *j* = 125 mA cm^{-2}	*ChemistrySelect* **2017,** *2,* 3401
**Cu_3P/CF**	1.0 M KOH + 0.5 M N_2H_4	0.72 V at *j* = 100 mA cm^{-2}	*Inorg. Chem. Front.* **2017,** *4,* 420
**Fe-CoS_2 nanosheets**	1.0 M KOH + 0.1 M N_2H_4	0.61 V at *j* = 100 mA cm^{-2}<br>0.95 V at *j* = 500 mA cm^{-2}	*This work*
	1.0 M KOH + 0.5 M N_2H_4	0.26 V at *j* = 100 mA cm^{-2}<br>0.42 V at *j* = 500 mA cm^{-2}	

Supplementary References

1. Zhang, J., Xiao, W., Xi, P., Xi, S., Du, Y., Gao, D. & Ding, J. Activating and optimizing activity of CoS_2 for hydrogen evolution reaction through the synergic effect of N dopants and S vacancies. *ACS Energy Lett.* **2,** 1022–1028 (2017).
2. Zhao, S., Jin, R., Song, Y., Zhang, H., House, S. D., Yang, J. C. & Jin, R. Atomically precise gold nanoclusters accelerate hydrogen evolution over MoS_2 nanosheets: the dual interfacial effect. *Small* **13,** 1701519 (2017).
3. Zhang, L., Han, L. L., Liu, H. X., Liu, X. J. & Luo, J. Potential-cycling synthesis of single platinum atoms for efficient hydrogen evolution in neutral media. *Angew. Chem. Int. Ed.* **56,** 13694–13698 (2017).
4. Li, H., Chen, S., Jia, X., Xu, B., Lin, H., Yang, H., Song, L. & Wang, X. Amorphous nickel-cobalt complexes hybridized with 1T-phase molybdenum disulfide via hydrazine-induced phase transformation for water splitting. *Nat. Commun.* **8,** 15377 (2017).
5. Tang, C., Zhang, R., Lu, W., Wang, Z., Liu, D., Hao, S., Du, G., Asiri, A. M. & Sun, X. Energy-saving electrolytic hydrogen generation: Ni_2P nanoarray as a high-performance non-noble-metal electrocatalyst. *Angew. Chem. Int. Ed.* **56,** 842–846 (2017).
6. Zhang, J. & Dai, L. Nitrogen, phosphorus, and fluorine tri-doped graphene as a multifunctional catalyst for self-powered electrochemical water splitting. *Angew. Chem. Int. Ed.* **55,** 13296–13300 (2016).

7. Yang, J., Wang, X., Li, B., Ma, L., Shi, L., Xiong, Y. & Xu, H. Novel iron/cobalt-containing polypyrrole hydrogel-derived trifunctional electrocatalyst for self-powered overall water splitting. *Adv. Funct. Mater.* **27,** 1606497 (2017).

8. Yin, J., Li, Y., Lv, F., Lu, M., Sun, K., Wang, W., Wang, L., Cheng, F., Li, Y., Xi, P. & Guo, S. Oxygen vacancies dominated NiS_2/CoS_2 interface porous nanowires for portable Zn–air batteries driven water splitting devices. *Adv. Mater.* **29,** 1704681 (2017).

9. Liang, H., Gandi, A. N., Anjum, D. H., Wang, X., Schwingenschlögl, U. & Alshareef, H. N. Plasma-assisted synthesis of NiCoP for efficient overall water splitting. *Nano Lett.* **16,** 7718–7725 (2016).

10. Mahmood, J., Li, F., Jung, S.-M., Okyay, M. S., Ahmad, I., Kim, S.-J., Park, N., Jeong, H. Y. & Baek, J.-B. An efficient and pH-universal ruthenium-based catalyst for the hydrogen evolution reaction. *Nat. Nanotech.* **12,** 441 (2017).

11. Zhang, R., Wang, X., Yu, S., Wen, T., Zhu, X., Yang, F., Sun, X., Wang, X. & Hu, W. Ternary $NiCo_2P_x$ nanowires as pH-universal electrocatalysts for highly efficient hydrogen evolution reaction. *Adv. Mater.* **29,** 1605502 (2017).

12. McKone, J. R., Sadtler, B. F., Werlang, C. A., Lewis, N. S. & Gray, H. B. Ni–Mo nanopowders for efficient electrochemical hydrogen evolution. *ACS Catal.* **3,** 166–169 (2013).

13. Liang, H.-W., Brüller, S., Dong, R., Zhang, J., Feng, X. & Müllen, K. Molecular metal–N_x centres in porous carbon for electrocatalytic hydrogen evolution. *Nat. Commun.* **6,** 7992 (2015).

14. Xiao, W., Liu, P., Zhang, J., Song, W., Feng, Y. P., Gao, D. & Ding, J. Dual-functional

N dopants in edges and basal plane of MoS_2 nanosheets toward efficient and durable hydrogen evolution. *Adv. Energy Mater.* **7,** 1602086 (2017).

15. Laursen, A. B., Patraju, K. R., Whitaker, M. J., Retuerto, M., Sarkar, T., Yao, N., Ramanujachary, K. V., Greenblatt, M. & Dismukes, G. C. Nanocrystalline Ni_5P_4: a hydrogen evolution electrocatalyst of exceptional efficiency in both alkaline and acidic media. *Energy Environ. Sci.* **8,** 1027–1034 (2015).
16. Popczun, E. J., Read, C. G., Roske, C. W., Lewis, N. S. & Schaak, R. E. Highly active electrocatalysis of the hydrogen evolution reaction by cobalt phosphide nanoparticles. *Angew. Chem. Int. Ed.* **53,** 5427–5430 (2014).
17. Cummins, D. R., Martinez, U., Kappera, R., Voiry, D., Martinez-Garcia, A., Jasinski, J.; Kelly, D., Chhowalla, M., Mohite, A. D., Sunkara, M. K. & Gupta, G. Catalytic activity in lithium-treated core–shell MoO_x/MoS_2 nanowires. *J. Phys. Chem. C* **119,** 22908–22914 (2015).
18. Jasion, D., Barforoush, J. M., Qiao, Q., Zhu, Y., Ren, S. & Leonard, K. C. Low-dimensional hyperthin FeS_2 nanostructures for efficient and stable hydrogen evolution electrocatalysis. *ACS Catal.* **5,** 6653–6657 (2015).
19. Gupta, S., Patel, N., Fernandes, R., Kadrekar, R., Dashora, A., Yadav, A. K., Bhattacharyya, D., Jha, S. N., Miotello, A. & Kothari, D. C. Co–Ni–B nanocatalyst for efficient hydrogen evolution reaction in wide pH range. *Appl. Catal. B: Environ.* **192,** 126–133 (2016).
20. Lee, R.-L., Tran, P. D., Pramana, S. S., Chiam, S. Y., Ren, Y., Meng, S., Wong, L. H. & Barber, J. Assembling graphitic-carbon-nitride with cobalt-oxide-phosphate to

construct an efficient hybrid photocatalyst for water splitting application. *Catal. Sci. Tech.* **3,** 1694–1698 (2013).

21. Li, Y., Yu, Y., Huang, Y., Nielsen, R. A., Goddard, W. A., Li, Y. & Cao, L. Engineering the composition and crystallinity of molybdenum sulfide for high-performance electrocatalytic hydrogen evolution. *ACS Catal.* **5,** 448–455 (2015).
