# Supplementary material for: Self-powered H2 production with bifunctional hydrazine as sole consumable
Source: Nat Commun. 2018 Oct 19;9:4365. doi: 10.1038/s41467-018-06815-9 (PMC6195518; doi:10.1038/s41467-018-06815-9)
Supplement: Supplementary file 3 — Description of Additional Supplementary Files [file 41467_2018_6815_MOESM3_ESM.pdf]

Description of Additional Supplementary Files

File Name: Supplementary Movie 1
Description: A working overall-hydrazine-splitting unit driven by an electrochemical workstation

File Name: Supplementary Movie 1
Description: A working self-powered overall-hydrazine-splitting system
